# YKL-40 and risk of incident cancer in early type 2 diabetes: a Danish cohort study

**DOI:** 10.1038/s41416-025-02996-5

**Published:** 2025-04-05

**Authors:** Alisa D. Kjaergaard, Allan A. Vaag, Verena H. Jensen, Michael H. Olsen, Kurt Højlund, Peter Vestergaard, Torben Hansen, Reimar W. Thomsen, Niels Jessen

**Affiliations:** 1 Steno Diabetes Center Aarhus, Aarhus University Hospital, Aarhus, Denmark; 2 Steno Diabetes Center Copenhagen, Copenhagen University Hospital, Herlev, Denmark; 3 Lund University Diabetes Center, Lund University, Lund, Sweden and Department of Endocrinology, Skåne University Hospital, Malmö, Sweden; 4 Department of Internal Medicine and Steno Diabetes Center Zealand, Holbæk Hospital, Holbæk, Denmark; 5 Steno Diabetes Center Odense, Odense University Hospital, Odense, Denmark; 6 Steno Diabetes Center North Denmark, Aalborg University Hospital, Aalborg, Denmark; 7 Novo Nordisk Foundation Center for Basic Metabolic Research, University of Copenhagen, Copenhagen, Denmark; 8 Department of Clinical Epidemiology, Aarhus University, Aarhus, Denmark

**Keywords:** Type 2 diabetes, Prognostic markers

## Abstract

**Background:**

We examined the association of serum YKL-40, an inflammatory biomarker, with incident cancer risk in early type 2 diabetes.

**Methods:**

A cohort of 11,346 individuals newly diagnosed with type 2 diabetes was followed for up to 14 years. YKL-40 levels (n = 9010) were categorised into five percentiles (0–33%, 34–66%, 67–90%, 91–95%, and 96–100%), and baseline YKL-40 and CRP (n = 9644) were analyzed continuously (per 1 SD log increment) for comparison. Cox regression models assessed associations with obesity-related, gastrointestinal, liver, pancreatic, colorectal, bladder and lung cancers, as well as cancers of reproductive organs.

**Results:**

Adjusted HRs (95% CIs) for the highest versus lowest YKL-40 category were 2.4 (1.6–3.7) for obesity-related, 2.6 (1.7–4.1) for gastrointestinal, 44.2 (12.8–153.4) for liver, and 4.2 (1.3–14.1) for bladder cancers. No associations were found for other cancers. YKL-40 and CRP had similar prognostic abilities for obesity-related and gastrointestinal cancers, but YKL-40 outperformed CRP for liver and bladder cancers. Conversely, CRP was a stronger predictor for lung, colorectal, and ovarian cancers.

**Discussion:**

YKL-40 was associated with the risks of liver and bladder cancers, clearly outperforming CRP for these cancers. This suggests distinct prognostic roles for YKL-40 and CRP, and highlights YKL-40 as a promising biomarker for liver cancer.

## Introduction

YKL-40, also known as chitinase-3-like protein 1 (CHI3L1), is an inflammatory biomarker produced locally at inflammation sites by cells such as tumour-associated macrophages and cancer cells [1]. Consequently, YKL-40 is elevated in many cancers, including liver, colorectal, and lung cancers, where it contributes to tumour progression by promoting growth, inflammation, immune evasion and metastasis [1, 2]. Consistent with this, inhibiting YKL-40 has been shown to effectively reduce tumour growth in these cancers [3–6].

Moreover, YKL-40 levels are consistently higher in individuals with type 2 diabetes compared to healthy controls [7, 8]. Given that obesity is a major modifiable risk factor for type 2 diabetes, there is a substantial overlap between the cancers associated with obesity (gastrointestinal, thyroid, and kidney cancers, multiple myeloma, meningioma, and breast, ovarian, and endometrial cancers) and those linked to type 2 diabetes [9, 10]. A meta-analysis of 32 million individuals found that type 2 diabetes is associated with increased risks of gastrointestinal (especially liver and pancreatic), thyroid, kidney, and bladder cancers, as well as breast, ovarian, and endometrial cancers in women, and a reduced risk of prostate cancer in men [9]. This overlap may arise from shared mechanisms, such as insulin resistance and chronic inflammation, that promote tumour development [11].

Prospective studies examining cancer risk have previously been conducted in the Danish general population, with cohorts including 8706 to 21,643 adults in the Greater Copenhagen area. These studies have shown that elevated YKL-40 is associated with an increased risk of developing gastrointestinal and possibly lung cancer, but not with breast, prostate, or other cancers [2, 12, 13].

However, to our knowledge, no prospective studies have ever specifically investigated YKL-40 levels in the context of type 2 diabetes and risk of cancer. Our study aimed to address this knowledge gap by assessing the prognostic value of YKL-40 in relation to cancers associated with obesity and type 2 diabetes in a contemporary cohort of individuals newly diagnosed with type 2 diabetes. Additionally, we compared the prognostic performance of YKL-40 to that of C-reactive protein (CRP).

## Methods

### The study cohort: DD2

The Danish Centre for Strategic Research in Type 2 Diabetes (DD2) cohort, established in 2010, is a nationwide collaborative project focusing on type 2 diabetes research [14]. Ongoing recruitment primarily occurs through general practitioners after a recent diagnosis of type 2 diabetes. Participants provide written consent and undergo a brief interview, physical exam, and contribute blood and urine samples stored in a biobank. Follow-up relies on national registry linkage. By April 2023, 11,346 participants were included, with YKL-40 and CRP biomarker levels measured in 9010 and 9644 individuals, respectively.

### YKL-40 levels

YKL-40 levels were determined using an electrochemiluminescence-based ELISA on the MESO Quickplex SQ 120 instrument (MSD, Gaithersburg, USA). The analysis utilised a U-plex assay within a custom multiplex panel [15]. Samples were measured once, with each plate containing a duplicate control pool, yielding a median intra-plate CV of 6.3% and an inter-plate CV of 18%.

### Covariates

Information on covariates was obtained from the enrolment interview and clinical examination or through linkage with the Danish Adult Diabetes Registry, a nationwide quality-of-care database. Additional information was sourced from the clinical laboratory information system, which has provided partial national coverage for laboratory tests since 2008 and complete coverage since 2016 (Supplementary Table 1). Laboratory results were used to supplement biomarker measurements, with only the closest values within six months of enrolment included.

A family history of type 2 diabetes was defined as having a first or second-degree relative with the condition (yes/no). Diabetes duration was calculated from diagnosis to enrolment (years). Body mass index (BMI) was derived from weight and height measurements (kg/m²), and the waist-to-hip ratio was determined by dividing waist by hip circumference. Alcohol overconsumption was classified based on weekly intake exceeding 14 standard drinks for women and 21 for men (yes/no). Smoking status was categorised as never or ever, and physical activity was classified as low (sedentary or light) or high (moderate to vigorous). Glycemic control was assessed by HbA1c, and revised homoeostasis model assessment (HOMA2) estimated β-cell function (HOMA2-B) and insulin sensitivity (HOMA2-S) as percentages of a normal reference population. The urinary albumin-to-creatinine ratio (UACR in mg/g) and estimated glomerular filtration rate (eGFR in mL/min/1.73 m^2^) were calculated, and low-density lipoprotein (LDL) cholesterol and triglycerides were expressed in mmol/L. C-reactive protein (CRP) levels in mg/L were measured using an in-house time-resolved immuno-fluorometric assay, as previously described [16].

### Outcomes

Our study aimed to investigate cancers associated with obesity and type 2 diabetes. The participants could have developed more than one cancer type during follow-up. Due to a limited incidence of some cancers, we focused our primary analysis on liver, pancreatic, colorectal, bladder, lung, breast, ovarian, and prostate cancers. To increase statistical power, we also examined grouped cancer categories: gastrointestinal cancers and obesity-related cancers. Gastrointestinal cancers (n = 329) included liver (n = 56), pancreatic (n = 46), and colorectal cancer (n = 204), as well as esophageal (n = 25), gastric cardia (n = 33), and gallbladder (n = 6) cancers. Obesity-related cancers (n = 362) encompassed these gastrointestinal cancers, along with thyroid (n = 4) and kidney (n = 25) cancers, as well as multiple myeloma (n = 6), and meningioma (n = 0). Additionally, we grouped breast (n = 141), ovarian (n = 46), and uterine (n = 15) cancers into a ‘female cancer’ category (n = 190).

We obtained diagnostic data, covering both primary and secondary discharge diagnoses, from the Danish National Patient Registry. Since 1977, this registry has systematically tracked all inpatient diagnoses, and, beginning in 1995, it expanded to include records from outpatient clinics and emergency departments. Supplementary Table 1 provides a list of the specific diagnosis codes used.

### Statistical analysis

Following the approach used in previous studies on cancer in the Danish general population [17–19], we stratified YKL-40 levels into five percentile categories: 0–33%, 34–66%, 67–90%, 91–95%, and 96–100%. Given that YKL-40 levels increase exponentially with age and differ between sexes [20], we calculated age- and sex-adjusted YKL-40 percentiles. This was done separately for each age group (18–50, 51–60, 61–70, and >70 years) stratified by sex, to ensure balanced age and sex distributions across the YKL-40 percentile categories (Table 1).

**Table 1:** Characteristics of the participants from the DD2 cohort of individuals newly diagnosed with type 2 diabetes according to age- and sex corrected YKL-40 percentile categories.

Covariates	Strata	Age and sex corrected YKL-40 percentile categories					
		0–33%	34–66%	67–90%	91–95%	96–100%	Overall
Number of participants		(N = 2975)	(N = 2970)	(N = 2160)	(N = 451)	(N = 454)	9010
Age, years		59.96 (11.37)	60.44 (11.58)	60.67 (11.54)	60.81 (11.40)	60.94 (11.85)	60.38 (11.51)
Sex							
	Female	1223 (41.1%)	1222 (41.1%)	889 (41.2%)	185 (41.0%)	187 (41.2%)	3706 (41.1%)
	Male	1752 (58.9%)	1748 (58.9%)	1271 (58.8%)	266 (59.0%)	267 (58.8%)	5304 (58.9%)
Family history of diabetes^a^							
	No^a^	1089 (36.6%)	1087 (36.6%)	876 (40.6%)	184 (40.8%)	199 (43.8%)	3435 (38.1%)
	Yes	1886 (63.4%)	1883 (63.4%)	1284 (59.4%)	267 (59.2%)	255 (56.2%)	5575 (61.9%)
Diabetes duration, years		1.78 (2.23)	1.78 (2.23)	1.83 (2.51)	1.80 (2.39)	1.97 (2.59)	2.04 (2.57)
Body mass index, kg/m²		30.37 (5.66)	30.37 (5.66)	31.45 (5.97)	32.74 (6.66)	33.11 (7.93)	32.54 (7.03)
	Missing	1020 (34.3%)	990 (33.3%)	691 (32.0%)	144 (31.9%)	147 (32.4%)	2992 (33.2%)
Waist-to-hip ratio		0.97 (0.09)	0.98 (0.09)	0.99 (0.08)	0.99 (0.09)	1.00 (0.08)	0.98 (0.09)
	Missing	33 (1.1%)	33 (1.1%)	29 (1.3%)	8 (1.8%)	6 (1.3%)	109 (1.2%)
Alcohol overconsumption^a^							
	No	2858 (96.1%)	2853 (96.1%)	1986 (91.9%)	393 (87.1%)	353 (77.8%)	8443 (93.7%)
	Yes	117 (3.9%)	117 (3.9%)	174 (8.1%)	58 (12.9%)	101 (22.2%)	567 (6.3%)
Smoking status							
	Never smoker	1055 (35.5%)	1028 (34.6%)	703 (32.5%)	132 (29.3%)	126 (27.8%)	3044 (33.8%)
	Ever smoker	1012 (34.0%)	997 (33.6%)	732 (33.9%)	184 (40.8%)	175 (38.5%)	3100 (34.4%)
	Missing	908 (30.5%)	945 (31.8%)	725 (33.6%)	135 (29.9%)	153 (33.7%)	2866 (31.8%)
Physical activity^a^							
	Low	2298 (77.2%)	2386 (80.3%)	1766 (81.8%)	372 (82.5%)	379 (83.5%)	7201 (79.9%)
	High	677 (22.8%)	584 (19.7%)	394 (18.2%)	79 (17.5%)	75 (16.5%)	1809 (20.1%)
HbA1c, mmol/mol		50.36 (11.38)	50.67 (11.32)	52.42 (12.58)	53.30 (13.67)	52.74 (12.62)	51.24 (11.90)
	Missing	507 (17.0%)	496 (16.7%)	322 (14.9%)	55 (12.2%)	46 (10.1%)	1426 (15.8%)
HOMA2-B, %		97.52 (43.62)	99.41 (43.60)	103.52 (46.04)	99.83 (46.92)	99.09 (44.94)	99.78 (44.49)
	Missing	181 (6.1%)	184 (6.2%)	127 (5.9%)	33 (7.3%)	41 (9.0%)	566 (6.3%)
HOMA2-S, %		41.92 (21.05)	38.32 (18.85)	34.79 (18.54)	34.93 (21.87)	32.99 (19.19)	38.23 (19.93)
	Missing	181 (6.1%)	184 (6.2%)	127 (5.9%)	33 (7.3%)	41 (9.0%)	566 (6.3%)
UACR, mg/g		26.55 (108.64)	39.21 (153.43)	56.18 (210.28)	59.11 (256.96)	183.93(1083.88)	48.11 (300.00)
	Missing	1220 (41.0%)	1143 (38.5%)	829 (38.4%)	166 (36.8%)	158 (34.8%)	3516 (39.0%)
eGFR, mL/min/1.73 m²		89.28 (16.16)	87.70 (18.18)	86.96 (19.63)	87.19 (20.10)	85.89 (22.34)	87.91 (18.31)
	Missing	734 (24.7%)	710 (23.9%)	466 (21.6%)	81 (18.0%)	78 (17.2%)	2069 (23.0%)
LDL cholesterol, mmol/L		2.32 (0.88)	2.32 (0.91)	2.28 (0.92)	2.28 (0.89)	2.30 (1.01)	2.31 (0.90)
	Missing	862 (29.0%)	870 (29.3%)	608 (28.1%)	125 (27.7%)	104 (22.9%)	2569 (28.5%)
Triglycerides, mmol/L		1.87 (1.66)	2.01 (1.41)	2.23 (1.69)	2.45 (1.61)	2.34 (1.67)	2.06 (1.60)
	Missing	874 (29.4%)	886 (29.8%)	608 (28.1%)	122 (27.1%)	104 (22.9%)	2594 (28.8%)
C-reactive protein, mg/L		2.73 (4.39)	3.85 (6.76)	4.83 (7.38)	6.21 (9.24)	6.65 (10.60)	3.97 (6.76)
	Missing	51 (1.7%)	50 (1.7%)	46 (2.1%)	<10 ( < 2.0%)	<10 ( < 2.0%)	159 (1.8%)
YKL-40, µg/L		21.51 (6.99)	41.32 (11.38)	77.98 (26.99)	144.89 (41.99)	328.60 (264.08)	63.23 (92.17)

Values were collected at (or close to) enrolment during 2010 through 2023, and expressed as numbers of participants (frequencies), or means (standard deviations).

*DD2* The Danish Centre for Strategic Research in Type 2 Diabetes, HbA1c glycated haemoglobin, HOMA2-B and HOMA2-S homoeostasis model assessment of β-cell function and insulin sensitivity, UACR urinary albumin-to-creatinine ratio, eGFR estimated glomerular filtration rate, LDL low density lipoprotein.

^a^Family history of diabetes was missing for 48 individuals, alcohol overconsumption for 29, and physical activity for 50. To address the issue of very small numbers (n < 5), i.e., microdata prohibited by the Danish Health Authority, missing values were, for this table only, re-coded to “No” for family history of diabetes, “No” for alcohol overconsumption, and “Low” for physical activity.

To compare the prognostic abilities of YKL-40 and CRP, we analyzed both biomarkers on a continuous scale (per one standard deviation [SD] increase in log-transformed levels) and by categorising DD2 participants into four groups: low levels of both YKL-40 and CRP, low YKL-40 with high CRP, high YKL-40 with low CRP, and high levels of both. We used cut-offs of <50 µg/L or ≥50 µg/L for YKL-40 (a rounded value between the mean and median) and <3 mg/L or ≥3 mg/L for CRP, the latter reflecting the traditionally accepted threshold for increased risk.

Hazard ratios (HRs) with 95% confidence intervals were estimated using Cox regression analysis with age as the time scale. The proportional hazards assumption was verified using Schoenfeld residuals, with no significant violations detected. Participants who experienced events before blood sampling, i.e., enrolment, were excluded from the analysis of the relevant outcome. We followed study participants from enrolment until the occurrence of an event, death, or October 27th, 2024, whichever occurred first. Recurrent events were not considered, and no adjustments were made for multiple comparisons.

We applied three increasingly detailed adjustments for potential confounders, to assess the robustness of the associations. Model 1 adjusted for age and sex. Model 2 built upon Model 1 by adding adjustments for family history of type 2 diabetes, type 2 diabetes duration, WHR, alcohol overconsumption and physical activity. Finally, Model 3 expanded on Model 2 by including additional adjustments for BMI, smoking status, HbA1c, HOMA2-B, HOMA2-S, UACR, eGFR, low density lipoprotein (LDL) cholesterol, and triglycerides.

In contrast to models 1 and 2, where complete case analyses were conducted without any imputation, model 3 employed multiple imputation to handle missing data for YKL-40, CRP, and covariates. This approach was chosen to increase statistical power and minimise potential selection bias. Imputation was performed using the *mice* (multivariate imputation by chained equations) R package version 3.16.0, with predictive mean matching applied to continuous variables (YKL-40, CRP, BMI, WHR, HbA1c, HOMA2-B, HOMA2-S, UACR, eGFR, LDL cholesterol, and triglycerides) and logistic regression to binary variables (family history of diabetes, alcohol overconsumption, smoking status, and physical activity) [21]. We generated 20 imputed datasets, with a maximum of 50 iterations, to ensure accurate data completion. The missing data distribution was consistent across YKL-40 categories at enrolment, with UACR having the highest missing values (39%), followed by BMI and smoking status (32–33%) (Table 1).

### Sensitivity analyses

To test robustness, we conducted several sensitivity analyses. First, study time was applied as the time scale to complement the age at enrolment approach used in the main analyses. Additionally, model 4 expanded on model 3 by including CRP as an extra adjustment variable, allowing us to assess whether the associations between YKL-40 percentile categories and outcomes were independent of systemic inflammation, as proxied by CRP. Lastly, we stratified analyses by dichotomised covariates, including age, sex, family history of diabetes, type 2 diabetes duration, BMI, WHR, alcohol overconsumption, smoking status, physical activity, HbA1c, HOMA2-S, HOMA2-B, UACR, eGFR, LDL cholesterol, triglycerides, and CRP. Due to limited events in some strata, we could only assess the risk per 1 SD increase in log(YKL-40) for obesity-related, gastrointestinal, liver, colorectal, and lung cancers, but not pancreatic and bladder cancers. For these analyses, we used a fully imputed dataset and adjusted for all covariates except the one stratified by.

## Results

We followed 11,346 participants from the DD2 cohort from 2010 to 2024. While the overall median follow-up time was nine years, the follow-up time specifically for cancer cases was generally four to five years, ranging from one year for pancreatic cancer to six years for liver cancer. During the follow-up period, 362 participants developed obesity-related cancers, of which 329 were gastrointestinal cancers, including 56 liver, 46 pancreatic, and 204 colorectal cancers. There were also 42 bladder cancer events and 78 lung cancer events. Additionally, there were 190 female cancer events in women (141 breast, 46 ovarian, and 15 uterine) and 202 prostate cancer events in men. Some participants developed multiple cancers.

### Characteristics of participants at DD2 enrolment

Table 1 shows the characteristics of participants at study enrolment, grouped by age and sex corrected YKL-40 percentiles (0–33%, 34–66%, 67–90%, 91–95%, 96–100%). Elevated YKL-40 levels were associated with longer diabetes duration, higher BMI and WHR, alcohol overconsumption, smoking, lower physical activity, higher HbA1c, lower insulin sensitivity (HOMA2-S), lower kidney function (albuminuria and lower eGFR), and higher triglycerides (but not LDL cholesterol) and CRP. Higher YKL-40 percentiles were linked to a lower frequency of family history of diabetes. HOMA2-B levels followed a bell-shaped pattern, peaking at 104% in the 67–90% YKL-40 category. The mean YKL-40 level in the overall cohort was 63 µg/L, increasing across percentiles from 22 µg/L to 329 µg/L (Table 1).

#### Risk of cancer across increasing YKL-40 percentile categories

**Fig. 1:**
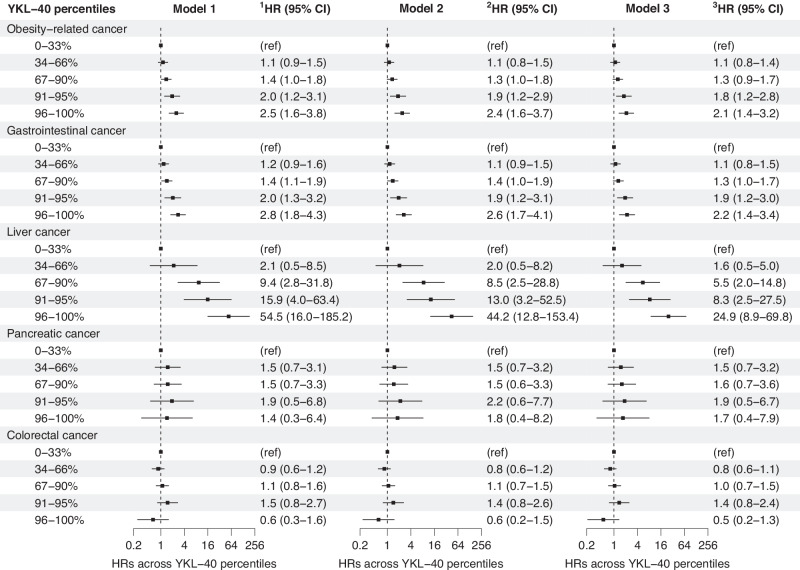
Risk of obesity-related cancers, including subtypes, across increasing YKL-40 percentile categories in individuals newly diagnosed with type 2 diabetes from the entire DD2 cohort. DD2: The Danish Centre for Strategic Research in Type 2 Diabetes. Obesity-related cancers included gastrointestinal (liver, pancreatic, colorectal, esophageal, gastric cardia, and gallbladder), thyroid and kidney cancers, as well as multiple myeloma and meningioma. Some individuals had multiple cancers. Cox regression, using age as time scale, estimated risks as hazard ratios (HRs) and 95% confidence intervals (CIs). 0–33% was the reference (ref) YKL-40 percentile category. Model 1 was adjusted for age and sex. Model 2 included additional adjustments for family history of type 2 diabetes, type 2 diabetes duration, waist-to-hip ratio, alcohol overconsumption and physical activity. In model 3, further adjustments were made for: body mass index, smoking status, HbA1c, HOMA2-B, HOMA2-S, urinary albumin-to-creatinine ratio, estimated glomerular filtration rate, low density lipoprotein cholesterol and triglycerides. Complete case analyses were used in models 1 and 2 (n = 9010), but model 3 (n = 11,346) employed multiple imputation to address missing data for YKL-40 and adjustment covariates, enhancing statistical power and reducing selection bias.

**Fig. 2:**
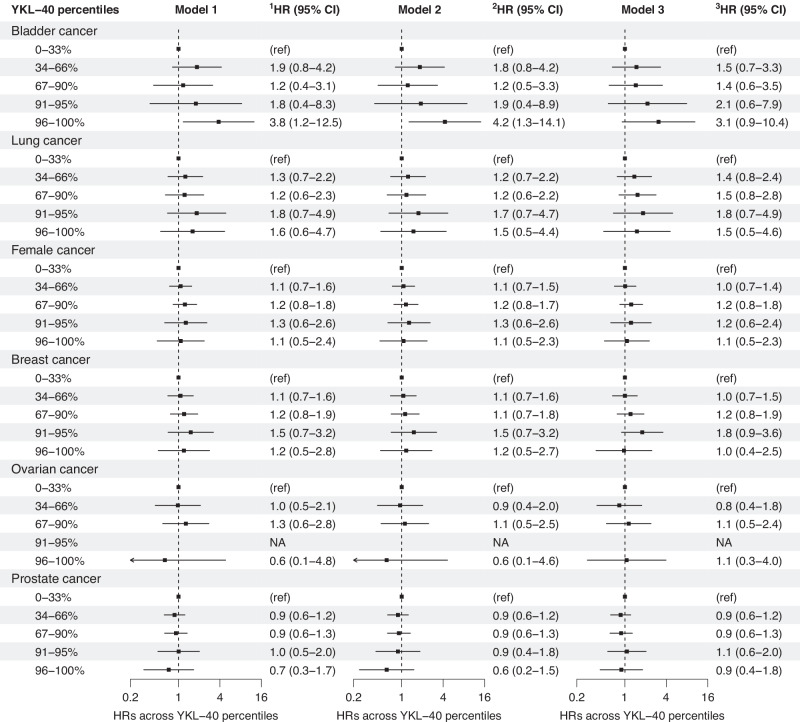
Risk of bladder, lung, and cancers of reproductive organs, across increasing YKL-40 percentile categories in individuals newly diagnosed with type 2 diabetes from the entire DD2 cohort. DD2: The Danish Centre for Strategic Research in Type 2 Diabetes. Female cancers included breast, ovarian and uterine cancers). Some individuals had multiple cancers. Cox regression, using age as time scale, estimated risks as hazard ratios (HRs) and 95% confidence intervals (CIs). 0–33% was the reference (ref) YKL-40 percentile category. Model 1 was adjusted for age and sex. Model 2 included additional adjustments for family history of type 2 diabetes, type 2 diabetes duration, waist-to-hip ratio, alcohol overconsumption and physical activity. In model 3, further adjustments were made for: body mass index, smoking status, HbA1c, HOMA2-B, HOMA2-S, urinary albumin-to-creatinine ratio, estimated glomerular filtration rate, low density lipoprotein cholesterol and triglycerides. Complete case analyses were used in models 1 and 2 (n = 9010), but model 3 (n = 11,346) employed multiple imputation to address missing data for YKL-40 and adjustment covariates, enhancing statistical power and reducing selection bias.

The risk of obesity-related and gastrointestinal cancers increased with higher YKL-40 percentile categories, primarily driven by liver cancer (Fig. 1, Supplemental Tables S2–S3). In model 2, adjusted HRs (95% CIs) for 96–100% versus 0–33% YKL-40 percentile category were 2.4 (1.6–3.7) for obesity-related cancers, 2.6 (1.7–4.1) for gastrointestinal cancers, 44.2 (12.8–153.4) for liver cancer, 1.8 (0.4–8.2) for pancreatic cancer, 0.6 (0.2–1.5) for colorectal cancer, 4.2 (1.3–14.1) for bladder cancer, and 1.5 (0.5–4.4) for lung cancer (Figs. 1–2, Supplemental Table 2). Corresponding estimates for cancers of reproductive organs were 1.1 (0.5–2.3) for female cancers, 1.2 (0.5–2.7) for breast cancer, 0.6 (0.1–4.6) for ovarian cancer, and 0.6 (0.2–1.5) for prostate cancer (Fig. 2, Supplemental Table S2). Further adjustments in models 3 and 4 led to a modest reduction in the risk estimates (Supplemental Tables S2–S3). Using an alternative time scale (time on study), produced similar results (Supplemental Tables S2–S3).

#### YKL-40 and CRP comparison

**Fig. 3:**
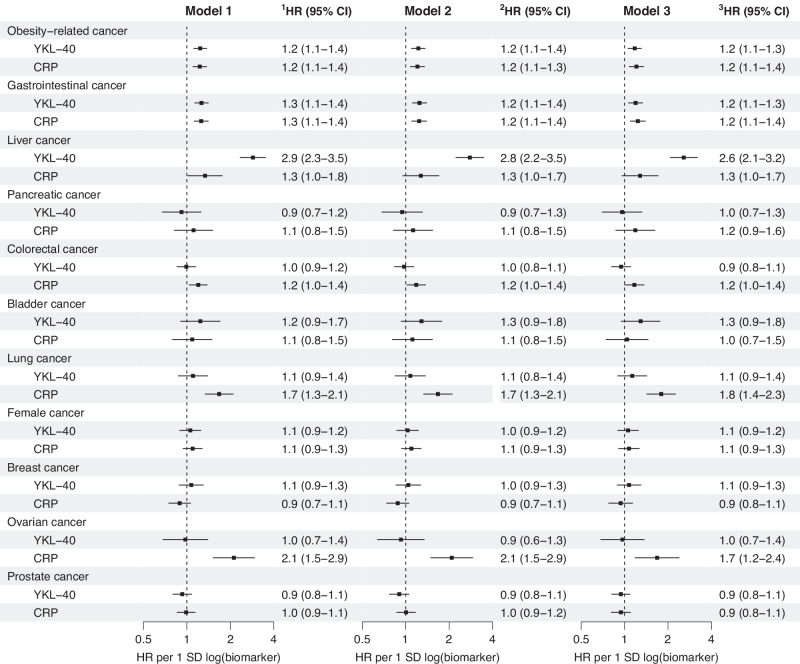
Risk of cancer per 1 SD log increase in YKL-40 and CRP levels, in individuals newly diagnosed with type 2 diabetes, from the entire DD2 cohort. DD2: The Danish Centre for Strategic Research in Type 2 Diabetes. NA: not available. Obesity-related cancers included gastrointestinal (liver, pancreatic, colorectal, esophageal, gastric cardia, and gallbladder), thyroid and kidney cancers, as well as multiple myeloma and meningioma. Female cancers included breast, ovarian and uterine cancers). Some individuals had multiple cancers. Cox regression, using age as time scale, estimated risks as hazard ratios (HRs) and 95% confidence intervals (CIs). Model 1 was adjusted for age and sex. Model 2 included additional adjustments for family history of type 2 diabetes, type 2 diabetes duration, waist-to-hip ratio, alcohol overconsumption and physical activity. In model 3, further adjustments were made for: body mass index, smoking status, HbA1c, HOMA2-B, HOMA2-S, urinary albumin-to-creatinine ratio, estimated glomerular filtration rate, low density lipoprotein cholesterol and triglycerides. Complete case analyses were used in models 1 and 2 (n = 9010 for YKL-40 and 9,644 for CRP), but model 3 (n = 11,346) employed multiple imputation to address missing data for YKL-40 and adjustment covariates, enhancing statistical power and reducing selection bias.

Each 1 SD log increment in YKL-40 and CRP levels resulted in similarly increased risk estimates for obesity-related and gastrointestinal cancers (Fig. 3, Supplemental Tables S4–S5). YKL-40 exhibited superior prognostic ability compared to CRP for liver and bladder cancers. Conversely, CRP demonstrated greater prognostic ability for lung cancer and possibly colorectal cancer, as well as ovarian cancer in women (Fig. 3 and Supplemental Tables S4–S5). This was confirmed by the joint analyses of YKL-40 and CRP based on low and high combinations of the two biomarkers (Supplementary Tables S6–S7). Risk estimates for obesity-related and gastrointestinal cancers for low YKL-40 and high CRP combination was similar to high YKL-40 and low CRP combination. In the presence of high YKL-40, liver cancer risk did not differ by low and high levels of CRP. Conversely, in the presence of high CRP, the risks of colorectal and lung cancers did not differ between low and high levels of YKL-40. Using different adjustments and an alternative time scale (time on study) yielded largely similar results (Supplemental Table S6–S7).

#### Stratified analyses

Stratified analyses by covariates, dividing into two groups each, revealed no significant differences in risk estimates, with 95% confidence intervals overlapping in all instances (Supplemental Table S8).

## Discussion

In this prospective study of over 11,000 individuals with newly diagnosed type 2 diabetes, elevated YKL-40 levels were a strong prognostic biomarker for obesity-related and gastrointestinal cancers, mainly driven by liver cancer. No associations were found with cancers of the pancreas, colorectum, lung, or reproductive organs. Elevated YKL-40 was strongly associated with increased risk of liver cancer and, to a lesser degree, bladder cancer, clearly outperforming CRP for these cancers. However, CRP demonstrated greater prognostic ability for lung, colorectal, and ovarian cancers.

This association may be due to reverse causation, where undiagnosed cancer raises YKL-40 and CRP levels. However, this does not explain all cancer events, as half occurred four to five years after enrolment. A more likely explanation is that inflammation, which increases both YKL-40 and CRP levels, could be the underlying cause, as both biomarkers are linked to cancer risk.

Consistent with previous studies, elevated YKL-40 levels were associated with obesity, alcohol overconsumption, smoking, and lower kidney function, as well as higher triglycerides and CRP, but not with LDL cholesterol [12, 18, 22, 23]. Additionally, elevated YKL-40 levels were associated with lower frequency of family history of diabetes, longer diabetes duration, higher HbA1c, and lower insulin sensitivity, as well as with lower physical activity, suggesting that very high YKL-40 levels may reflect lifestyle rather than genetic factors.

Previous Danish general population studies (<10% had diabetes) have shown that elevated YKL-40 is associated with an increased risk of developing gastrointestinal cancers and possibly lung cancer, but not with breast or prostate cancers [2, 12, 13]. In these studies, YKL-40 was a stronger prognostic marker for gastrointestinal cancers, while CRP showed better prognostic ability for lung cancer. While our findings largely align with these observations, our study could not support the lung cancer association, possibly due to limited statistical power. Similarly, the associations for pancreatic and colorectal cancers in our study had risk estimates above 1.0, although they did not differ from 1.0. As previous studies did not distinguish individual cancers within the gastrointestinal group, direct comparisons with our findings on liver, pancreatic and colorectal cancers are not possible. However, for liver cancer specifically, we find supportive evidence from similar risk estimates observed for alcoholic liver cirrhosis within the previous Danish population cohorts: the HRs for the highest compared to the lowest YKL-40 category were strikingly similar, at 44 in our liver cancer study and 41 in a previous alcoholic liver cirrhosis study [23]. This alignment likely reflects the shared risk factor of alcohol overconsumption, which was notably more prevalent in the highest YKL-40 category (22%) compared to the lowest (4%). Thus, YKL-40 may serve as a non-invasive alternative to liver biopsy for identifying individuals at high risk of alcohol-related liver disease, potentially enhancing treatment and adherence to alcohol reduction. While this potential needs further testing, using plasma YKL-40 could reduce costs, minimise patient morbidity, and avoid liver sampling errors.

This is also the first prospective study to investigate the association between elevated baseline YKL-40 levels and the risk of incident bladder cancer. A prior study has shown that YKL-40 levels are higher in 67 patients with bladder cancer compared to 65 controls and correlate with disease severity [24].

Despite only 46 ovarian cancer events in 4642 women, we found a strong association between elevated baseline CRP levels and increased ovarian cancer risk. This is consistent with findings from a UK Biobank study of 232,908 women, including 1110 ovarian cancer events, which identified a trend of rising ovarian cancer risk with increasing CRP quartiles [25]. Nevertheless, the results for cancers of reproductive organs may be impacted by limited power due to stratification. Indeed, for prostate cancer, the risk estimate for elevated YKL-40 levels was below 1.0, indicating a reduced risk, following a similar effect pattern to that observed for type 2 diabetes [9].

### Strengths and limitations

The key strengths of this study include its large cohort of individuals newly diagnosed with type 2 diabetes, comprehensive clinical and lifestyle data, and reliable, high-quality, population-based health registries with complete follow-up. Another strength of this study is the use of multiple imputation, which reduces selection bias by utilising all available data instead of excluding participants with missing values. This enhances the reliability of the results, particularly when data are not missing completely at random.

Potential limitations include survival and selection bias, which may have led to underrepresentation of individuals with severe type 2 diabetes or high cancer risk, likely biasing results toward the null hypothesis. Additionally, the use of registry-based diagnoses introduces the possibility of misclassification, though any such errors are likely random and would tend to underestimate the true association. Participants who developed cancer shortly after enrolment were not excluded from the analyses, which could introduce bias through reverse causation, where undiagnosed cancer elevates YKL-40 levels. However, this cannot explain all cancer events, as approximately half occurred four to five years after enrolment. For liver cancer specifically, half of the events occurred even later, around six years post-enrolment.

## Conclusions

In conclusion, studying more than 11,000 individuals newly diagnosed with type 2 diabetes, we found that elevated YKL-40 levels were strongly associated with increased risk of liver cancer, and to a lesser extent, bladder cancer, clearly outperforming CRP for these cancers. However, CRP demonstrated superior prognostic ability for lung, colorectal, and ovarian cancers. These findings indicate that YKL-40 and CRP have distinct prognostic roles in different cancer types among individuals with type 2 diabetes. YKL-40, in particular, shows strong precision medicine potential as a biomarker for liver cancer, supporting its use in early detection and focused monitoring in this population.

## Supplementary information

Supplementary tables 1-8

## Data Availability

The datasets analyzed during the current study are not publicly available due to privacy and GDPR concerns. Information about the procedure for data access is available on the cohort website: dd2.dk.

## Code availability

Statistical analyses were conducted using R version 4.3.3 on a secure cloud server at the Danish Health Data Authority, and are not publicly available due to privacy and General Data Protection Regulation (GDPR) concerns.
